# Towards OPM-MEG in a virtual reality environment

**DOI:** 10.1016/j.neuroimage.2019.06.010

**Published:** 2019-06-04

**Authors:** Gillian Roberts, Niall Holmes, Nicholas Alexander, Elena Boto, James Leggett, Ryan M. Hill, Vishal Shah, Molly Rea, Richard Vaughan, Eleanor A. Maguire, Klaus Kessler, Shaun Beebe, Mark Fromhold, Gareth R. Barnes, Richard Bowtell, Matthew J. Brookes

**Affiliations:** a Sir Peter Mansfield Imaging Centre, School of Physics and Astronomy, University of Nottingham, University Park, Nottingham, NG7 2RD, United Kingdom; b QuSpin Inc. 331 S 104th St, Louisville, CO, 80027, USA; c Aston Laboratory for Immersive Virtual Environments, School of Life and Health Sciences, Aston University, Birmingham, BE4 7ET, United Kingdom; d Wellcome Centre for Human Neuroimaging, UCL Queen Square Institute of Neurology, University College London, 12 Queen Square, London, WC1N 3AR, United Kingdom; e Aston Brain Centre, School of Life and Health Sciences, Aston University, Birmingham, BE4 7ET, United Kingdom

## Abstract

Virtual reality (VR) provides an immersive environment in which a participant can experience a feeling of presence in a virtual world. Such environments generate strong emotional and physical responses and have been used for wide-ranging applications. The ability to collect functional neuroimaging data whilst a participant is immersed in VR would represent a step change for experimental paradigms; unfortunately, traditional brain imaging requires participants to remain still, limiting the scope of naturalistic interaction within VR. Recently however, a new type of magnetoencephalography (MEG) device has been developed, that employs scalp-mounted optically-pumped magnetometers (OPMs) to measure brain electrophysiology. Lightweight OPMs, coupled with precise control of the background magnetic field, enables participant movement during data acquisition. Here, we exploit this technology to acquire MEG data whilst a participant uses a virtual reality head-mounted display (VRHMD). We show that, despite increased magnetic interference from the VRHMD, we were able to measure modulation of alpha-band oscillations, and the visual evoked field. Moreover, in a VR experiment in which a participant had to move their head to look around a virtual wall and view a visual stimulus, we showed that the measured MEG signals map spatially in accordance with the known organisation of primary visual cortex. This technique could transform the type of neuroscientific experiment that can be undertaken using functional neuroimaging.

## 1. Introduction

In a typical functional neuroimaging experiment, a participant is asked to lie with their head at the centre of a fixed imaging system. They are exposed repeatedly to stimuli designed to evoke brain activity whilst data are continuously recorded; subsequent data processing allows inference on the location, magnitude, and time-course of the evoked brain activity. This technique has revolutionised neuroscience by enabling a noninvasive window on the working human brain, in health and disease. However, a major limitation is that most neuroimaging instrumentation requires the participant to maintain a fixed head position throughout the experiment. This introduces major limitations on the type of experiment that can be carried out. For example: it has been difficult to study the neural underpinnings of behaviours like spatial navigation, where head movement (to look at one’s surroundings) is an integral part of the task. Similarly, examining some aspects of social interaction is precluded due to the unnatural environment in which the participant is placed. In particular, because stimulus presentation is generally limited to simple 2D visual scenes, it is difficult to place individuals in an immersive environment that can be used to probe high level function (and dysfunction). These are just some examples of the ways in which current generation of neuroimaging technology limits addressable neuroscientific questions. In this paper, we aim to show that these significant limitations might be lifted by the combination of quantum technology, magnetoencephalography (MEG), and virtual reality (VR).

MEG (Cohen, 1968) measures the small (femtoTesla-scale) magnetic fields that are generated outside the head by neural currents in the brain. In this way, human brain electrophysiology can be measured with good (~3–5 mm) spatial resolution (Barratt et al., 2018) and excellent (~1 ms) temporal precision. In recent years, new computational algorithms for mathematically modelling MEG data (Gross et al., 2001; Robinson and Vrba, 1998) have led to a marked increase in its utility, and MEG has been shown to provide unique insights into fundamental neuroscientific questions; for example, allowing elucidation of the critical role played by neural oscillations in the formation and dissolution of the brain networks that support cognition (Baker et al., 2014; Brookes et al., 2011; O’Neill et al., 2015, 2017). Unfortunately, MEG technology itself is limited: conventional systems employ sensitive superconducting quantum interference devices (SQUIDs) (Hämäläinen et al., 1993) which can measure magnetic fields on a scale of ~10 fT, but the requirement for superconductivity means that these sensors must be housed within a cryogenic dewar. This means sensor positions are fixed within an immobile (one--size-fits-all) cryogenic helmet; sensors are consequently located 2–3 cm from the scalp, lowering measurable signal. Moreover, participant movement relative to the sensors degrades data quality, and paradigms requiring large head movements are impossible. However, recent developments in quantum technology have led to the introduction of new sensors known as optically-pumped magnetometers (OPMs) (Kominis et al., 2003). These sensors exploit the endogenous spin properties of alkali metals to measure magnetic fields with a similar sensitivity to SQUIDs, but without the need for cryogenic cooling. A number of studies have demonstrated the applicability of OPMs in MEG (Borna et al., 2017; Boto et al., 2018; Johnson et al., 2013; Kamada et al., 2015) and recent developments have seen the introduction of small (Sander et al., 2012) and lightweight commercial OPMs (Osborne et al., 2018), which can be mounted on the scalp. Since the external surface of these sensors is approximately at body temperature, they can be brought within ~6–8 mm of the scalp surface, leading to a significant increase in the measurable signal (Boto et al., 2016; Iivanainen et al., 2017).

A significant problem with scalp-mounted MEG sensors is sensitivity to the ambient magnetic field. Almost all MEG experiments are conducted inside a magnetically-shielded room (MSR) – an enclosure surrounded by multiple layers of high permeability (mu) metal which ensures a magnetically “quiet” environment. However, in most shielded rooms used for MEG there is a residual (temporally) static magnetic field of order 20–30 nT. This means that a scalp-mounted OPM, moving (with the head) relative to this field, will detect a signal much larger than that related to brain activity; indeed the signal is sufficiently large that even a small movement (e.g. 4° of head rotation in a 25-nT field) is enough to take an OPM outside its dynamic range and render it inoperable (Boto et al., 2018; Holmes et al., 2018). For this reason, background fields must be eliminated if OPMs are to realise their potential in offering a wearable imaging technology. This has been the topic of recent work (Boto et al., 2018; Holmes et al., 2018; Iivanainen et al., 2018), which has shown that appropriately designed electromagnetic coils can be deployed to generate fields equal and opposite to the remnant background Earth’s field, thereby cancelling it out and introducing a ‘null space’ around the participant’s head. This has led to novel experiments in which MEG data have been recorded in participants undertaking natural tasks such as drinking, playing a ball game (Boto et al., 2018), or even rotating their head to shift a visual scene to different parts of the visual field (Holmes et al., 2018). It follows that small, lightweight OPMs, in combination with precise magnetic field control, offer a new opportunity to acquire high fidelity neuroimaging data in moving participants, and the potential for completely novel experimental paradigms.

Virtual reality allows the user to feel presence within an environment mediated by technology providing sensory input (Steuer, 1992). This can be achieved using a head mounted display (HMD) or computer automated virtual environment (CAVE – where the display is mounted on surfaces surrounding the user) systems. These work based upon two principles: first, two images are projected independently to the user’s eyes; these images show the same scene, but shifted spatially in order to mimic the parallax induced by interpupillary distance. This gives the impression of viewing a 3-dimensional (3D) scene. Second, by tracking the position and orientation of the user’s head, the image shown to the eyes can be updated in real time. For the participant, this means that they can move their head in order to visually explore their environment. This phenomenon, called motion parallax, is very powerful in providing depth and stereo cues to the observer, promoting the impression of full immersion in a 3D world.

Virtual reality technologies are becoming popular tools for psychology research in areas such as social interaction (Pan and Hamilton, 2018), immersion therapy (Carl et al., 2019) and episodic memory (La Corte et al., 2019). As virtual reality becomes more mainstream in research it is important that we are able to use existing neuroimaging methods to complement it. From a neuroimaging perspective, VR is attractive since it allows a participant to be placed into almost any (virtual) environment imaginable, but in a controlled manner where careful temporal management of events can be maintained (enabling, for example, data averaging). The current technique of choice for combining with VR is electroencephalography (EEG) which has been successfully used to measure brain activity elicited by VR stimuli (Tromp et al., 2018). However, even high density EEG (hd-EEG) suffers from relatively poor spatial resolution, compared to MEG, due to the inhomogeneous conductivity profile of the skull which makes the EEG forward problem hard to model (Baillet, 2017). Moreover, EEG data are contaminated by artifacts caused by electrical activity in muscles of the head and neck. This is particularly problematic when head movement is allowed (or encouraged) during VR use (Boto et al., 2018; Muthukumaraswamy, 2013). MEG is approximately 10 times less susceptible to interference from muscles in the neck and head. Further, even conventional (cryogenic) MEG has significantly better spatial resolution than EEG, and the use of OPMs offers further (fundamental) improvements. For these reasons, the development of VR-MEG has significant advantages over the current generation of technology.

Here, we describe the use of a VRHMD system in combination with a recently developed OPM-MEG instrument, to measure brain activity evoked by a VR environment. Specifically, we aimed to: (1) demonstrate that, even with the VRHMD in place, OPMs were sensitive to brain activity via measurement of alpha-band neural oscillations in the occipital lobe; (2) use the same instrument to measure visual evoked activity (which is smaller in magnitude than alpha oscillations, thus posing a greater challenge) (3) exploit the properties of VR in a paradigm in which a participant was asked to move their head to view a previously occluded visual stimulus.

## 2. Methods

### 2.1. OPM-MEG system overview

**Fig. 1.**
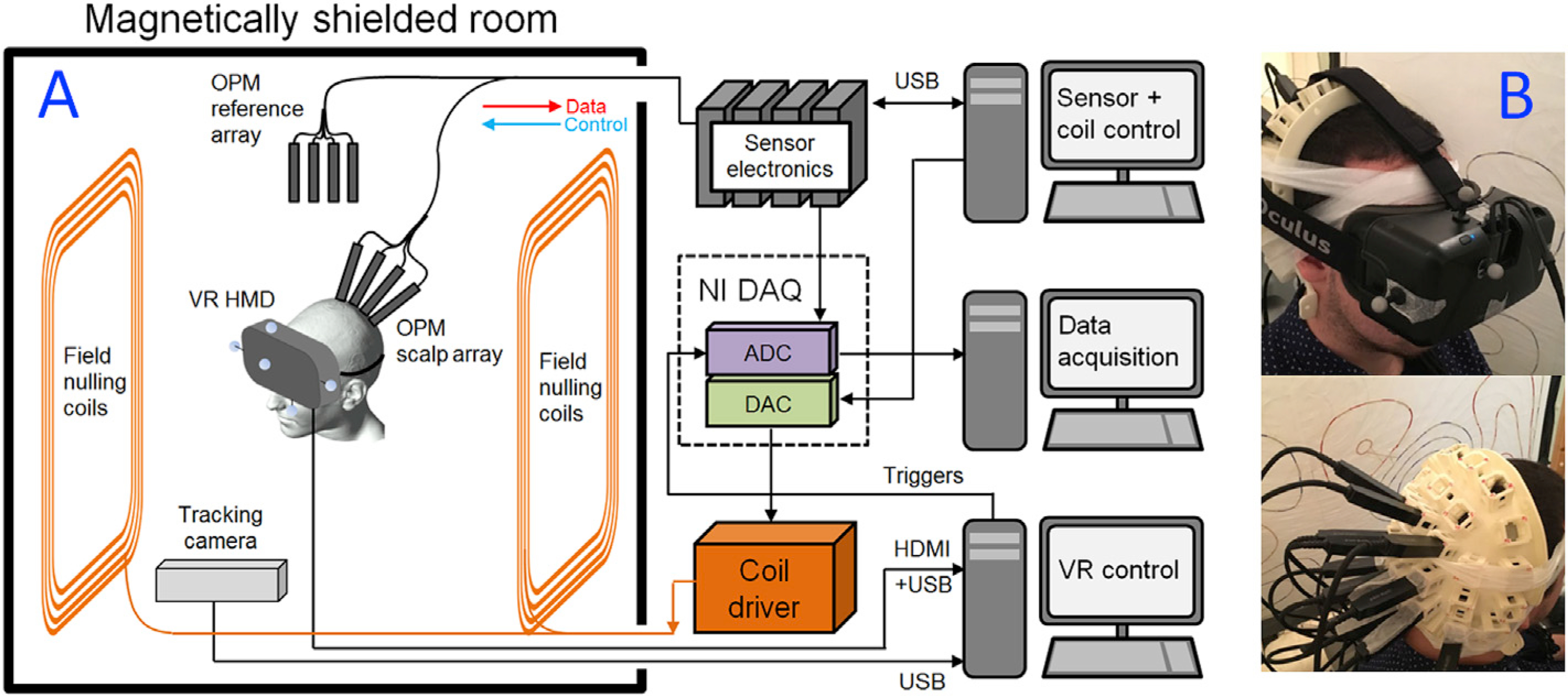
VR-OPM-MEG system overview. (A) A schematic overview of the complete system. (B) VRHMD placed on a participant, with OPMs mounted in slots in a 3D-printed scanner-cast, which was moulded to fit the back of the head. Note OPMs are placed over the visual areas.

We used the prototype OPM-MEG system, depicted schematically in Fig. 1A (Boto et al., 2018; Holmes et al., 2018; Tierney et al., 2018). An array of OPMs (QuSpin Inc., Louisville, CO) was placed in a 3D-printed scanner-cast (Boto et al., 2017) which was mounted over the visual cortex. OPMs were mounted in a bilaterally symmetric pattern over the visual cortex, with the maximally inferior OPM placed at the inion. A further 4 OPMs were placed in a reference array around 20 cm away from the head. Prior to MEG recording, the reference array OPMs were used to measure the background (static) magnetic field inside the MSR, and a feedback loop was used to control current through a set of bi-planar nulling coils (Holmes et al., 2018). Consequently, we could reduce the background field in a 40 × 40 × 40-cm^3^ region surrounding the head, thus enabling free head movement during scanning. The VRHMD was mounted over the participant’s eyes, and controlled by a separate computer. The VR control computer was also used to send triggers to the data acquisition computer, to denote the start or end of stimulation and, therefore, enable data processing. A tracking camera (Opti-Track V120:Duo, NaturalPoint Inc.) was used to passively measure head movement (via IR reflectors attached to the VRHMD, see Fig. 1), and this information was fed into the VR computer to allow updating of the visual scene that the participant saw in accordance with their head movement. All control equipment was kept outside the MSR to reduce interference.

### 2.2. OPMs

OPMs exploit the spin properties of alkali atoms and optical pumping to generate a measure of local magnetic field. Each OPM sensor head contains a 795-nm wavelength semiconductor laser for optical pumping, optics for laser beam conditioning, a 3 ×3 × 3-mm^387^Rb vapour cell and a silicon photo-diode for beam detection. The sensor head connects to a small electronics controller which sits outside the MSR. Optical pumping moves ^87^Rb atoms into the so-called ‘dark’ quantum state and, in the absence of an external field, they cannot escape, or absorb further photons. Thus, the atomic vapour becomes transparent to laser light. However, in the presence of an external field, atoms escape this state, and begin absorbing photons, meaning the vapour opacity increases. This manifests as a zero-field resonance with high sensitivity to small external fields. Here, we employed compact sensors manufactured by QuSpin Inc. Each sensor includes three on-board coils which can be used to null any remnant static field components in the cell, thereby enabling the zero-field resonance. The intensity of light transmitted through the cell is a Lorentzian function of the magnetic field component transverse to the laser beam, with a full width at half maximum of around 30 nT. For continuous field measurements, a sinusoidally-modulated magnetic field of ~1 kHz frequency was applied, perpendicular to the laser beam, using the on-sensor coils. The depth of modulation of the transmitted light, which is monitored using a lock-in process, is sensitive to the magnitude of the field component along the modulation axis. The amplitude of the two field components perpendicular to the beam can be measured simultaneously by applying oscillating currents to two coils in quadrature. However, here only the radial field component was measured.

### 2.3. Field nulling

For the VRHMD we used a consumer-grade Oculus Rift Development Kit 2 (Oculus VR LLC, Menlo Park, CA.), which was mounted over the eyes. This system was modified by removal of ferromagnetic screws, but a number of ferromagnetic components that were capable of generating a static magnetic field across the head, remained. In order for the OPMs to work, this field (like any static background field) must be removed necessitating a modification of the approach to field nulling described in previous studies (Boto et al., 2018; Holmes et al., 2018).

To understand the modified field-nulling process, we separate the background field into two components, the remnant Earth’s field in the room, BE and the field due to the VRHMD, ***B_H_***: the total background field is ***B_T_*** = ***B_E_*** + ***B_H_***. Importantly, ***B_E_*** and ***B_H_*** differ in their characteristics: BE is defined relative to the MSR; ***B_H_*** is defined relative to the VRHMD (and hence the head). This means that in the reference frame of the OPMs on the participant’s head, *B_H_* will not change in time (since the static field moves with the VRHMD, and therefore with the head). *B_E_* will change in time as the head moves relative to the MSR. Consequently, different nulling methods are required to cancel these two fields.

In order to cancel ***B_E_*** we employed the bi-planar coils, which were able to generate three components of static field (*B_x_*, *B_y_* and *B_z_*) as well as three components of field gradient ($\frac{dB_{x}}{dz}$, $\frac{dB_{y}}{dz}$ and $\frac{dB_{z}}{dz}$), with all fields generated relative to the MSR. Prior to introduction of the VRHMD, we measured ***B_E_*** using our reference array and then cancelled it using the bi-planar coils. The currents in the bi-planar coils were then held constant until after the experiment was complete.

In the presence of the VRHMD and with the bi-planar coils switched on (i.e. with ***B_E_*** → 0) a static field measurement yields an estimate of ***B_H_*** at each OPM. The on-sensor coil currents were then set to optimally cancel this prior to the experiment starting. Since ***B_H_*** is constant relative to the head, these currents could be calculated at the start of the experiment and then held constant, with head movement having minimal effect.

### 2.4. Virtual reality

VR environments were designed using the Unreal Engine 4 SDK (version 4.17) (Epic Games, Inc.), a freely available game engine with developer tools which permit the integration of a VR headset to display a simulated game environment to players. The SDK has a visual scripting language which allows the designer to control the behaviour of objects in the simulation. To send 5 V trigger signals from the parallel port (and hence to the acquisition computer), it was necessary to integrate parallel port driver libraries into the Unreal project. This was achieved using open source code written by Logix4U (http//www.highrez.co.uk/downloads/inpout32/). In this way, eight independent trigger channels could be controlled by events in the VR simulation. These triggers were read by the data acquisition ADC channels.

The Oculus Rift was modified such that spatial tracking was achieved, not by the electromagnetically active infrared LEDs integrated into the headset (as is usually the case in standard operation), but via an OptiTrack V120: Duo dual camera infrared (IR) system that tracked passive IR-reflective markers mounted onto the VRHMD. This helped reduce magnetic interference measured at the OPMs. Head tracking was performed using the MotiveTracker software alongside a NaturalPoint plugin for streaming real-time motion-tracking data directly to Unreal Engine 4. Five IR-reflective marker balls were attached to the Oculus Rift (see Fig. 1). By illuminating these balls with an integrated IR light source, the OptiTrack was able to triangulate the position of the markers at a rate of 120 Hz and with sub-millimetre precision. A rigid body was defined in the tracking software, with two markers defining the interpupillary axis through their placement on opposite sides of the headset. This enabled definition of a VR environment, without the need for IR LEDs on the headset itself.

### 2.5. Data collection

OPM-MEG data were collected during three experiments. We expected that operation of the VRHMD in close proximity to the OPMs would generate significant interference (the majority of which we believe to be caused by current loops related to pixel switching in the screen that provides the visual scene; see Supplementary Material Fig. 6. For these reasons, the first two experiments were designed to test whether sufficient signal-to-noise ratio to measure MEG data could be realised. In the third experiment, we aimed to show that our OPM system could cope with the head movement that is required to fully exploit a VR environment. These studies were approved by the University of Nottingham Medical School’s Ethics Committee and all participants gave written informed consent.

#### 1) Alpha oscillations:

Neural oscillations at the alpha frequency (8–13 Hz) (Berger, 1929) are among the largest electrical signals recorded from the brain in MEG. Here, we aimed to show that the OPMs could detect modulation in alpha amplitude, despite electromagnetic interference. Ten OPMs were mounted over the occipital lobe. The experimental paradigm comprised 100 s during which the participants looked at a virtual (3D) visual scene, and 100 s when their eyes were closed (and the VRHMD displayed a black screen). The scene was stationary, but head-tracked, so head movement would change the aspect seen by the participant. However, participants were instructed to sit still, but without any requirement to visually fixate on a particular part of the scene presented. This experiment was carried out in ten participants (mean age 31 ± 11 years, 8 male, 2 female) and we expected to see an increase in alpha-band oscillatory amplitude on closing the eyes.

#### 2) Visual evoked field:

The visual evoked field is robustly elicited when participants watch a reversing checkerboard pattern (Shigeto et al., 1998). Nevertheless, its amplitude is lower than that of alpha waves. Here we measured MEG data whilst participants watched a reversing checkerboard pattern, again using an array of 10 OPMs sited over the visual cortex. The checkerboard had 8^2^ subdivisions and was reversed at a frequency of 0.86 Hz. The checkerboard was presented as part of a virtual scene, to both eyes in stereoscopic format. The stimulus included a red fixation dot in the centre of the checkerboard which was present throughout the whole experiment (i.e. even when the checkerboard was not visible). Eleven participants took part in the study (mean age 31 ± 11 years, 9 male, 2 female). We expected to see a visual evoked response on each of the 180 reversals of the checkerboard. However, we also expected that the VRHMD would generate a stimulus-locked artefact, since the current in loops in the VRHMD screen must change when the pixels in the checkerboard change from black to white. We reasoned that these currents would generate a measurable magnetic field which would average constructively across checkerboard cycles. For this reason, we also recorded MEG data using the same stimulus, but with the OPMs and VRHMD mounted on a phantom (a polystyrene head).

#### 3) Head movement and visual cortex topology:

Here we aimed to undertake a more realistic VR experiment that required head movement. Twelve OPMs were mounted on the head over the visual cortex. The participant was presented with a visual scene in which they were placed behind a virtual wall. By leaning to their right or left, they were able to look around the wall, at which point they were able to see a reversing checkerboard, which was part of the distant visual scene. The experiment comprised 80 trials (40 leaning left, 40 leaning right), each of 15-s duration. At the beginning of each trial the participant was instructed to lean either to the left or to the right, around the wall, and gaze at a fixation dot. As they moved, a reversing checkerboard (8^2^ divisions, reversing at 4 Hz) appeared and this was displayed for 3 s. Following this, there was 3 s of rest after which the subject was instructed to move back behind the wall. Trials where the participant leaned left and right were interleaved. Importantly, in trials where the participant moved right, the visual stimulus appeared to the left of the fixation dot. Similarly in trials where the participant moved left, the visual stimulus appeared to the right of the fixation dot. In this way, the checkerboard primarily stimulated the left visual field on left-leaning trials, and the right visual field on right-leaning trials. We expected that the 4 Hz flashing stimulus would generate a response at 8 Hz, that would be mapped laterally in primary visual cortex due to optical decussation (i.e. we would observe a response in the left hemisphere when the participant leaned left and a response in the right hemisphere when the participant leaned right). A single participant (male, 23 years old) took part in the study, and they were scanned 3 times to assess consistency. The effect of magnetic interference was once again assessed by performing the same experiment on a phantom. The VR scene was altered to remove the wall, so that the stationary phantom was exposed to the screen-related magnetic effect of the inverting checkerboard.

### 2.6. Data analysis

Following data collection, we adopted a gradiometer approach to data processing in which signals from pairs of neighbouring magnetometers were subtracted, forming five synthetic channels which approximated planar gradiometers. This was done to reduce common mode interference generated by the VRHMD. For the visual cortex topology experiment, we used 12 magnetometers and expanded the gradiometer set to include all nearest neighbours.

For the ***alpha oscillation experiment***, data were segmented into two epochs of 100 s; the first during the period where the participants’ eyes were open, and the second during the eyes-closed time window. A frequency spectrum was computed (using the absolute value of the Fourier transform) for each segment and these spectra were averaged (independently for each condition) over participants. We tested for a significant increase of oscillatory amplitude over the alpha-band (8–13 Hz) in the eyes-closed condition, using a non-parametric Wilcoxon sign rank test, corrected for multiple comparisons across the 5 gradiometer signals using false discovery rate (FDR) correction (Benjamini and Hochberg, 1995).

For the ***visual evoked field experiment***, gradiometer signals were averaged over each reversal of the checkerboard pattern, yielding a single time course, 1.16 s in duration, for each gradiometer. We expected to see a deflection in the first 100 ms corresponding to the visual evoked field. We therefore measured the variance in the 0–100-ms window and compared this to variance in the 100–200-ms window; this was calculated for all gradiometers, and all 11 participants. We tested for significance again using a Wilcoxon sign rank test and corrected for multiple comparisons using FDR correction.

For the ***visual cortex topology*** experiment we employed a more complex analysis, based upon that described previously by Holmes et al. (2018). Our aim was to demonstrate that the expected hemispheric differences in visual cortex response could be mapped spatially, and to this end we employed a beamformer analysis (Brookes et al., 2008; Robinson and Vrba, 1998; Van Veen et al., 1997).

A requirement of beamforming is that one needs accurate knowledge of the sensor locations relative to brain anatomy; here this was provided using a procedure described in Zetter et al. (2019). The 3D printed headcast gave accurate knowledge of the OPM locations relative to each other and so only the location of the cast relative to the head was required. For this we used a Kinect V1 depth camera (Microsoft) in conjunction with Skanect 3D scanning software (Occipital Inc.) to generate a digital rendering of the surface of the participant’s head and face. Image data were stitched together to generate a 3D point cloud representation of the participant’s head, with approximately 700,000 vertices. One scan was taken with the participant’s hair covered by a swimming cap, to approximate the scalp head shape reconstructed from a structural magnetic resonance imaging (MRI) brain scan. A second optical scan was taken immediately before the experiment, with the participant wearing the scanner-cast. Co-registration was performed by surface matching the two optical scans, first to each other, and then to the scalp surface extracted from the participant’s structural MRI (the MRI scan had previously been acquired using a Phillips 3T Ingenia MRI scanner, with a T1-weighted gradient echo sequence and a voxel size of 1 mm. A high bandwidth was used to reduce distortion (Liuzzi et al., 2017; Meyer et al., 2017)).

Following co-registration a beamformer was applied to the data; the forward solution was computed using a single-sphere head model and a dipole approximation, using the analytical formulation first described by Sarvas (1987). Gradiometer data were bandpass-filtered from 4 to 12 Hz using a 4th-order Butterworth filter. We constructed a trial average covariance using a time window spanning the duration of stimulus presentation (checkerboard) and rest (i.e. 6 s of (averaged) data in total). The covariance matrix was then regularised using the Tikhonov method with a regularisation parameter equal to 5 percent of the maximum singular value of the un-regularised matrix. We contrasted oscillatory power in the 0 s-3 s time window (i.e. during the checkerboard) with the equivalent oscillatory power in the 3 s-6 s time window (i.e. during rest) to generate pseudo-t-statistical images showing the spatial distribution of the response. This was computed independently for trials in which the participant leaned left or right, yielding two images in which we expected to see responses in left and right primary visual cortex respectively. Finally, we derived “virtual sensor” signals from the peaks in the pseudo-t-statistical images, and Fourier transformed them to test for the presence of 8 Hz peaks. Gradiometer time courses were also derived by averaging over trials.

## 3. Results

**Fig. 2.**
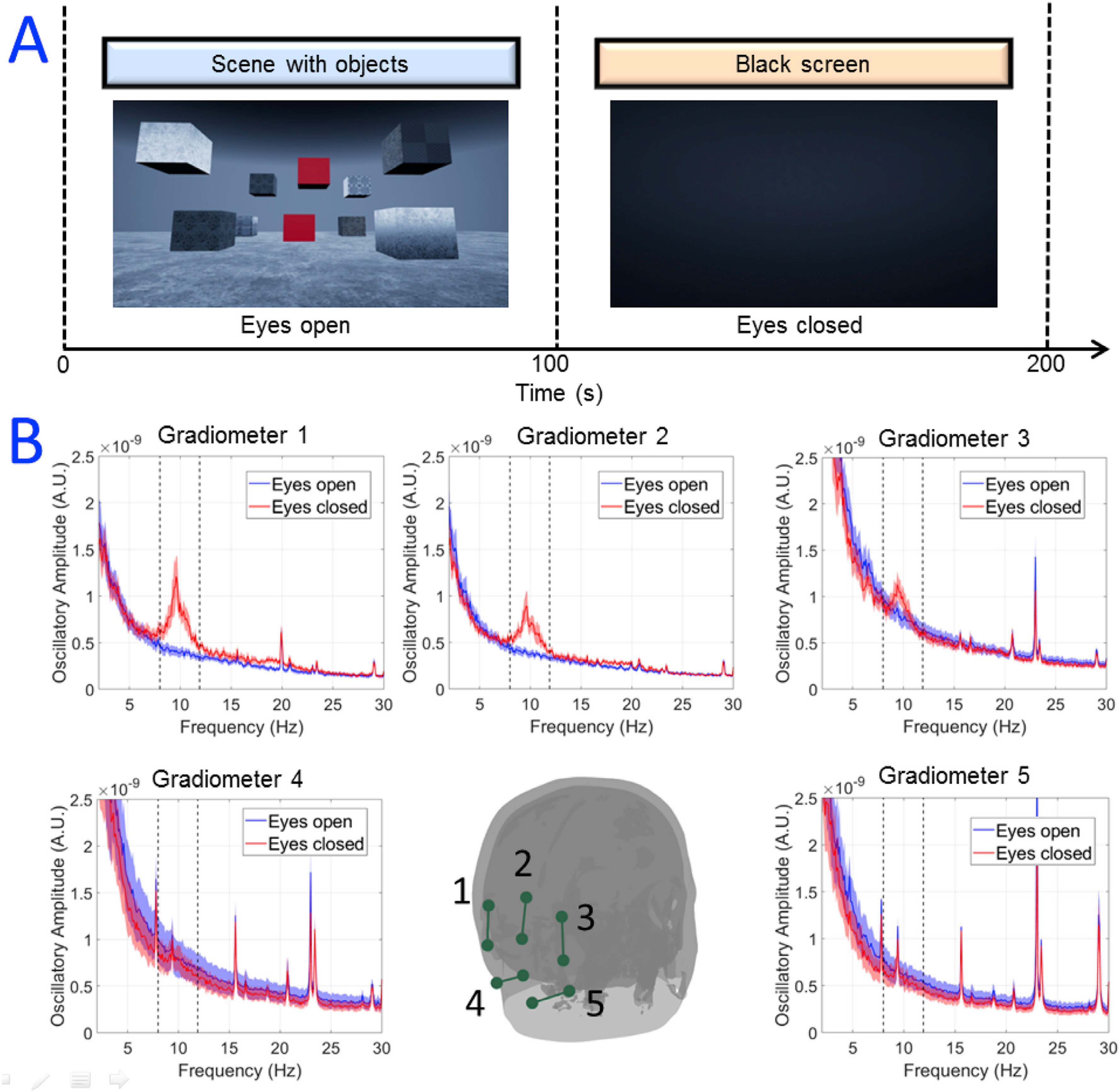
Modulation of alpha oscillations. (A) Schematic diagram showing the paradigm in which a static 3D visual scene was presented to the participants for 100 s whilst they had their eyes open, and then faded to black when the participants closed their eyes for a further 100 s. (B) OPM measurements - the coloured dots show locations of the OPM sensors on the scalp. The 10 magnetometers were formed into 5 gradiometer channels marked by the dark green lines. The inset graphs show power spectra of the measured data, averaged over 10 participants. Red shows data with eyes closed and blue shows data with eyes open. The shaded area shows the standard deviation over participants. Note the significant increase in alpha oscillations when the eyes were closed, in gradiometer channels sensitive to visual cortex.

Fig. 2 shows the results of the alpha-band experiment. Despite the increased magnetic interference caused by the VRHMD, we observed a statistically significant (p = 0.01 - non-parametric sign rank test) modulation in alpha oscillations, with smaller amplitude in the eyes-open (with visual stimulus) case than in the eyes-closed case. These results show clearly that MEG signals can be measured in the presence of a VRHMD showing a static 3D scene.

**Fig. 3.**
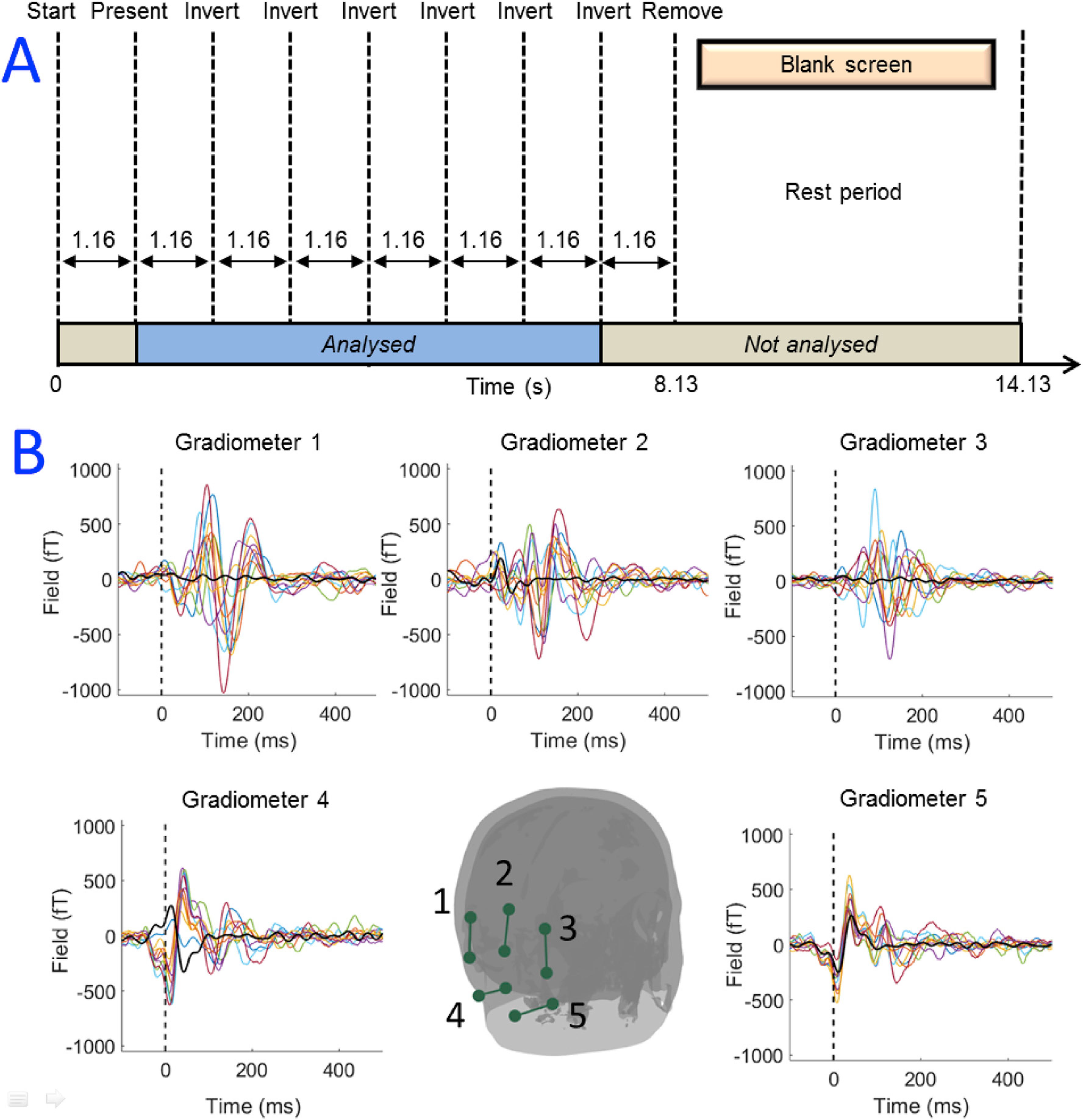
Visual evoked response experiment. (A) Schematic diagram of the experiment. (B) OPM measurements - the dots show locations of the OPM sensors on the scalp. The 10 magnetometers were formed into 5 gradiometer channels indicated by the green lines. The inset graphs show gradiometer time courses for all participants, averaged over 180 checkerboard reversals. Black shows data from the phantom.

Fig. 3 shows results from the visual evoked response experiment. We point out that the checkerboard stimulus itself, when sent to the VRHMD, did generate significant magnetic interference with deflections in the signal measurable even in the phantom experiment. However, these artifacts were smaller than the signals from the cortex, as can be seen by comparison of the blue and red traces in Fig. 3B. Clear visual evoked responses were observed, across multiple gradiometer channels, in 10 out of 11 participants. One participant’s evoked field data was excluded from the average due to an unidentified persistent artifact with a standard deviation at least five times the peak amplitude of the largest evoked response (consequently, the evoked response was not observed in this participant). The grand average and standard deviation of all other participants is shown in the supplementary materials (Fig. 8). Statistical testing showed that across the group, signal variance in the first 100 ms following checkerboard reversal was significantly (p = 0.01) larger than that in the 100 ms–200 ms window. This suggests that despite the relatively high levels of magnetic interference generated by the VRHMD showing a reversing checkerboard, MEG signals were clearly measurable.

**Fig. 4.**
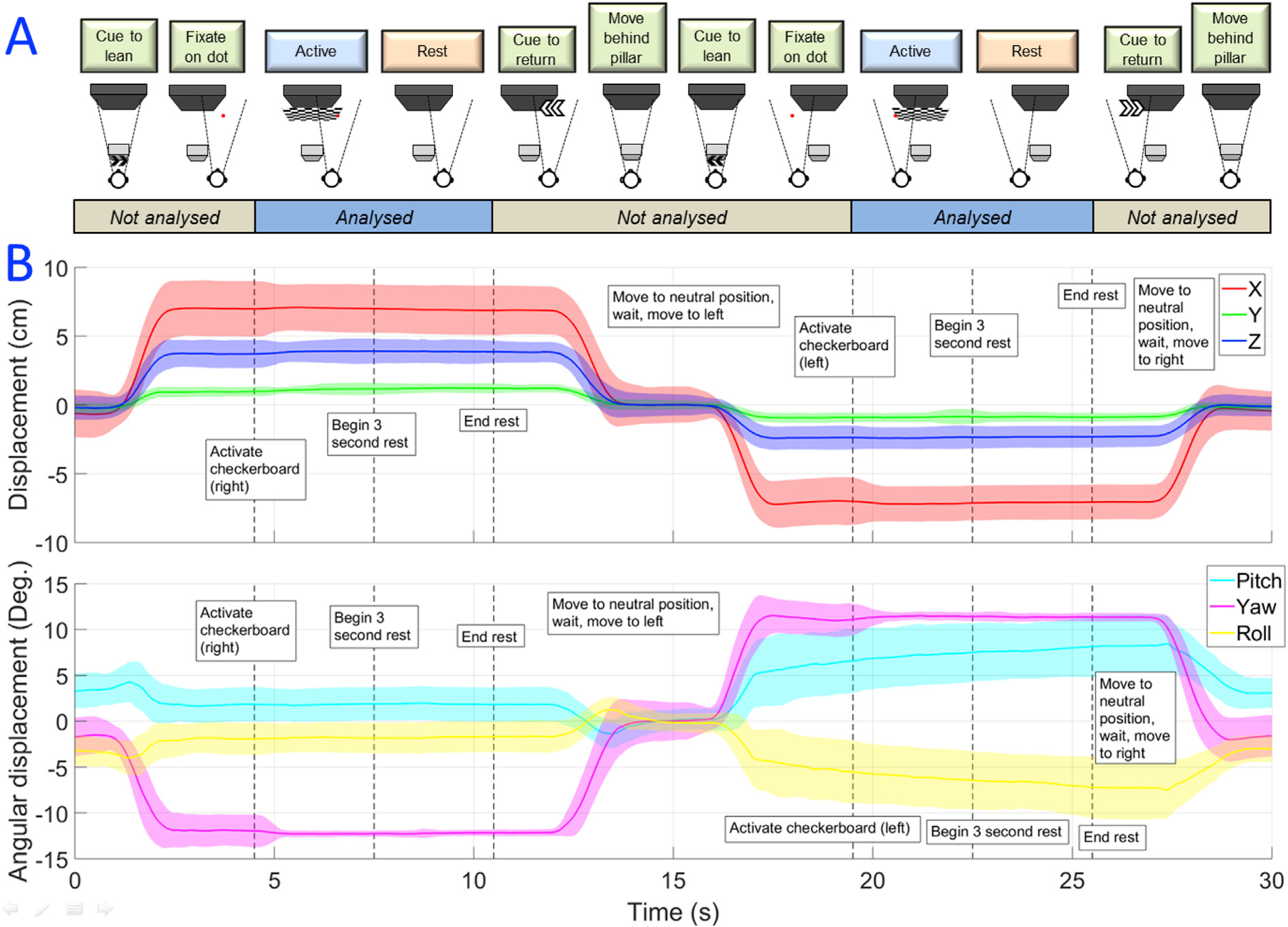
The visual cortex topology experiment - movement. (A) Schematic diagram showing the task. (B) The scale of movement, with translation in x (left-right), y (up-down) and z (forward-backwards) shown in the upper panel, and rotations about x, y and z shown in the lower panel. Standard deviations for each variable are represented by the shaded areas. Note the large movements required to complete the task, which could not be carried out using a conventional neuroimaging technique.

Fig. 4A shows the results of the visual cortex topology experiment. On average, the participant moved from −7±2 cm to +7 ±2 cm on each pair of trials (based on measurement along the axis with the greatest movement). This was accompanied by a head rotation from −12 ± 2° to +12 ± 2°. It is noteworthy that head movements on this scale could not be performed in conventional imaging systems including both cryogenic MEG and (functional) MRI (fMRI).

**Fig. 5.**
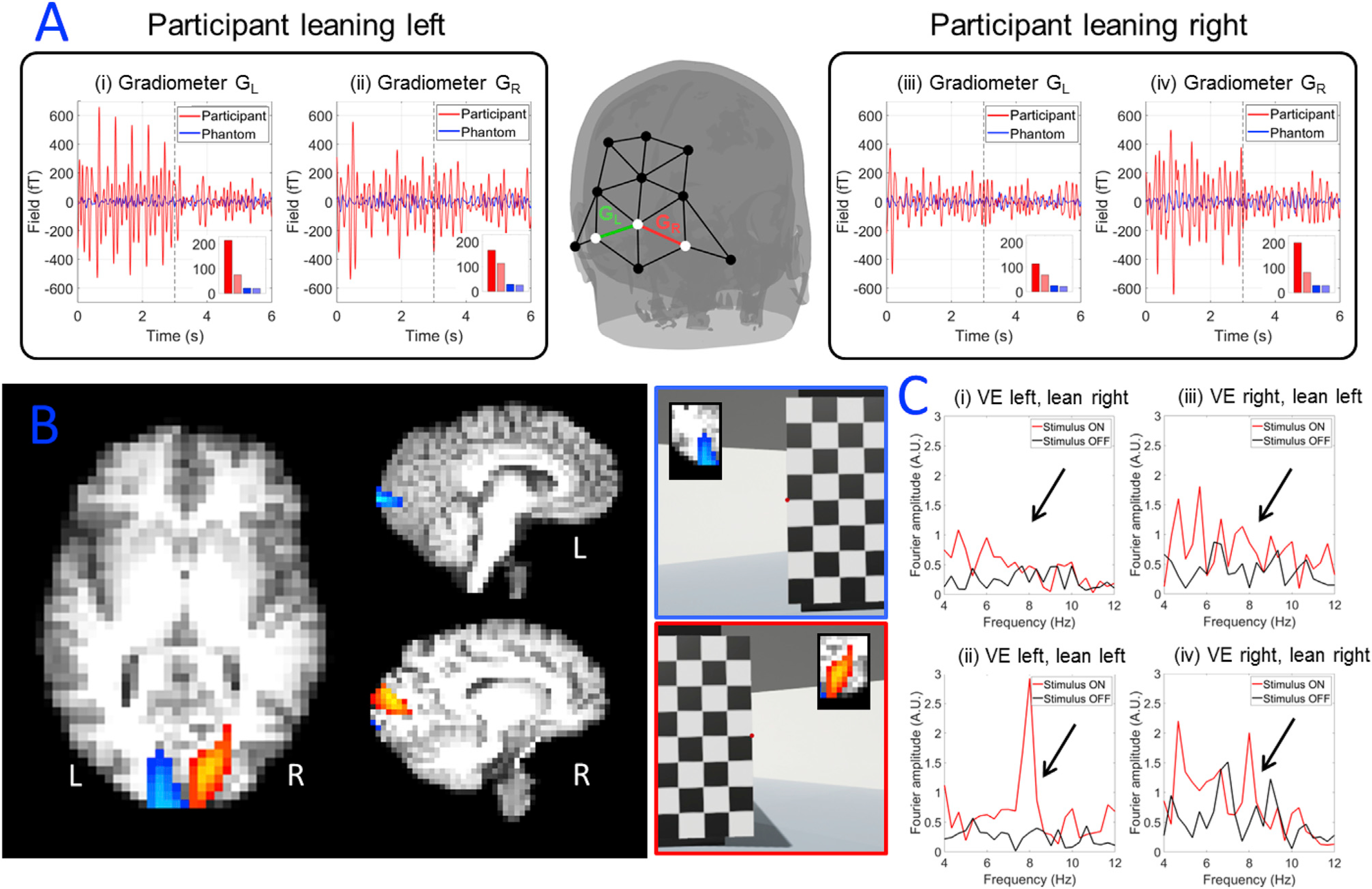
MEG results of visual cortex topology from a representative run. (A) Trial averaged gradiometer traces showing magnetic fields measured. (i) and (ii) show gradiometer over left (i) and right (ii) hemisphere with the participant leaning left. (iii) and (iv) show gradiometer over left (iii) and right (iv) hemisphere with the participant leaning right. A bar graph is inset in each gradiometer trace, showing the signal’s standard deviation for the participant in the active (red) and rest (light red) windows. The bar graph also shows standard deviation for the phantom, again in the active (blue) and rest (light blue) windows. Note the response to the alternating checkerboard in the first 3 s of stimulation in the case of contralateral visual stimulation of the participant. (B) Beamformer pseudo-T-statistical images showing the spatial signature of the largest 8 Hz modulation; the blue overlay shows the case where the participant is leaning to the left (and so the visual stimulus appears on the right); the red overlay shows the case where the participant is leaning to the right (and so the visual stimulus appears on the left). Note the hemispheric separation of responses: (C) frequency spectra of beamformer-reconstructed time-courses, extracted from peaks in the pseudo-T-statistical images. (i) and (ii) show left hemisphere with the participant leaning right (i) and left (ii). (iii) and (iv) show right hemisphere with the participant leaning left (iii) and right (iv).

Fig. 5 shows the MEG results of this experiment. Oscillatory responses can be seen in the first three seconds of the gradiometer data, corresponding to the checkerboard presentation. Beamformer pseudo-tstatistical images were produced showing the spatial signature of 8-Hz modulation, overlaid onto axial and sagittal slices of the participant’s anatomical scan. The images show strong contralateral activation in response to stimulation, in accordance with the well-known spatial organisation of the visual cortex.

## 4. Discussion

The introduction of movement-enabled VR-based stimuli to functional neuroimaging would potentially offer a step change in paradigm design with significant consequences for systems neuroscience. Currently, visual stimulation is typically limited to presenting 2-dimensional scenes – while this offers some flexibility, it is difficult to truly immerse a participant in a particular task or environment. VR technology would allow the use of more realistic experimental paradigms, enabling neuroscientists to ask new questions about brain function. For example, being able to move through virtual worlds will greatly advance the study of spatial navigation. The ability to place someone in a stressful environment might allow us to understand more about how the brain deals with pressure, and how decision making is affected. A number of organisations (e.g. police and military forces) now use VR as part of their training and concurrent measures of brain activity might inform our understanding of how individuals learn to cope with specific roles or tasks. VR therapy is also used in a number of different domains (e.g. treatment for post-traumatic stress disorder, PTSD) and understanding how the brain responds to such treatments might offer significant new insights into treatment efficacy.

In this paper, we have provided a proof-of-concept that OPM-MEG offers a viable option as a functional brain imaging technique that can be coupled with VR. The key point is that, for VR to work properly, participants must be allowed to make free movements of the head, and this largely rules out fMRI, positron emission tomography (PET), or conventional MEG, all of which rely on compliant participants maintaining an approximately static head position. There are alternative approaches to wearable brain imaging including EEG and functional nearinfrared spectroscopy (fNIRS). However, EEG lacks spatial precision and is highly susceptible to artifact generated by muscles in the head and neck during head movement. Given that we wanted to encourage such movement here, EEG becomes compromised. fNIRS is a useful technique in which brain haemodynamics are inferred when probed with nearinfrared light. However, the limitation brought about by indirect (blood-based) measurement means poor temporal resolution; further, spatial resolution is also limited to ~10 mm. For these reasons, OPM-MEG offers the best compromise of high spatiotemporal resolution whilst enabling a participant to move.

Importantly, the range of movement allowed here is limited only by the bi-planar coils that we employed. Specifically, this particular set of coils enables free movement within a 40 × 40 × 40-cm^3^ cube surrounding the head – whilst sufficient for many applications, this might limit some paradigms. However, the limitation is based only upon the size of the coils, which in turn is based on the practicalities of the MSR in which they are sited (in the case of the present work, the MSR also houses a conventional cryogenic MEG system which significantly limited the size of the coils that we could build). The available space to move could therefore be increased by building larger coils. This would enable even greater flexibility of movement.

The principal problem with VR-OPM-MEG, as described here, is interference at the OPMs generated by the VRHMD. The majority of this interference is generated by the internal OLED display - as pixels update, a current loop from the pixel to the screen origin is activated which generates a magnetic field. The further the pixel is from the origin, the larger the loop and hence the larger the artifact. In addition, pixel brightness and colour also impact the artifact size. Consequently, the interference depends on what the VRHMD is actually displaying. Here, noise recordings (see Supplementary Material Fig. 6) showed that mean interference in the 8–13 Hz band (for magnetometers/gradiometers) was 17/24 fT/√Hz in the absence of the VRHMD; 52/23 fT/√Hz when the VRHMD showed a stationary image; 217/28 fT/√Hz when the VRHMD showed a flashing checkerboard, and 211/35 fT/√Hz when it showed a video. Given the unpredictable nature of head movement, and consequently the unpredictable nature of the pixel display in VR, the OLED screen produces a rapidly-changing magnetic field pattern which is hard to predict, and consequently the interference is difficult to cancel.

Nevertheless, we have shown that MEG data can be recorded in the presence of interference. Our alpha-band demonstration represents a simple example in which the VRHMD showed a visual scene. Pixels would have only updated during the 100 s of the eyes-open condition in cases where the participant made an appreciable head movement; in the absence of such head movements, the pixel values will remain static and so the interference is minimised. This demonstration consequently constitutes the best possible scenario in terms of signal-to-interference ratio, with relatively little interference and perhaps the largest electrophysiological signal in the brain. Our results showed a clear alpha peak in 3 of the 5 gradiometers formed, with the other 2 likely being positioned too far towards the base of the subject’s neck to capture real alpha oscillations. It is noteworthy that, in the three gradiometers that did measure an alpha peak, there appeared to be some variability in peak height and width. This, we believe, was caused by a changing baseline interference (i.e. in Fig. 2B, the baseline noise in gradiometer 3 is appreciably higher than gradiometers 1 and 2). Whilst the reason for this is unclear, it is likely due to either the location and/or orientation of the sensors with respect to each other (meaning that the gradiometer is less effective) or a high baseline noise in a single OPM. In future studies, better positioning of OPMs to form planar gradiometers in which the orientation of the two sensors is equivalent might ameliorate such effects. Nevertheless, it is compelling that neurophysiological effects in MEG data could be measured in the presence of a VRHMD, and potentially this technique could explore, for instance, differences in processing of 2D/3D visual scenes.

The reversing checkerboard represented a more challenging situation in terms of signal-to-interference ratio. Here, pixel values were turned from black to white (the largest change they can undergo) and this was time-locked to the expected modulation of the neuromagnetic field. We reasoned that this would give one of the largest artifacts. Nevertheless, as evidenced by results in Fig. 3, using synthesized gradiometers we were able to observe significant stimulus-induced activity from the brain, with the visual evoked field measurable. These responses compare well with those shown in previous literature (Shigeto et al., 1998) (for a direct comparison, see Supplementary Material Fig. 8) in terms of both temporal morphology, and peak latencies. Importantly, as shown by our phantom data in Fig. 3, the peaks due to brain activity (which occur ~100/150 ms post stimulation) are separated in time from artifacts due to the VRHMD artifact (which occurs ~50 ms), providing more confidence that OPMs were measuring real brain activity. We do note a hemispheric discrepancy, with the field measured over right hemisphere being smaller in amplitude than that over left hemisphere and the reason for this is unknown. We calculated the standard deviation of the field in the 300 ms post-stimulus period in the left and right gradiometers for all participants, and found the difference between left and right to be significant using a paired *t*-test (p = 0.02). However this could be an effect of partial field cancellation caused by the stereoscopic nature of the stimulation. Nevertheless, the strong agreement in latency and morphology suggest that a genuine neurophysiological response is measurable.

The most challenging experiment was our visual cortex topology study which combined artifacts from the VRHMD with significant subject movement on the scale of 15 cm translations and 30° rotations. Here, we saw that, even in the presence of a changing visual stimulus (again, white to black pixels within the checkerboard) we were able to detect brain responses that mapped to the expected area of visual cortex. This provides significant evidence that even this simple set-up can generate usable MEG data. This potentially offers the possibility of implementing interesting visual experiments even at this early stage of VR-MEG development.

Despite our positive results, interference remains a major issue with the current experimental design; indeed this would likely become worse if OPM sensors were brought into closer proximity to the VRHMD. Here, the VRHMD was mounted at the front of the head and the OPMs at the back, and so we were in the best possible position to record artifact-free activity. However, measurement in the frontal lobes would likely pose a greater challenge – both because of the increased interference (which is likely to change as 1/r^3^) and also because of ferromagnetic material causing a large offset field which cannot be cancelled by the OPMs’ on-board sensor coils. It is therefore important that future VR-MEG studies should treat the VR-OPM-MEG method with caution, since stimulusrelated artifact generated by the OLED screen could unwittingly be interpreted as brain activity. Here, we employed phantom experiments to measure the artifact due to a reversing checkerboard, and showed temporal separation between the artifact and the neurophysiological response. In addition, in our spatial mapping experiment, we used the known topological functional anatomy of the visual cortex to show that the reconstructed signals are being generated by the expected brain regions (which, of course, would be extremely unlikely if measurable signals were generated by artifact). However these are not the only methods to rule out interference from artifacts. For example, asking participants to close their eyes while presenting VR stimulation may be a way to measure artifacts without real brain activity. This has the advantage that, unlike the phantom experiment, the subject would be able to move, enabling measurement of any artifacts of movement (e.g. including muscle artifact) without the VR stimulation. Additionally, VR offers predefined “camera positions” which can change over time, which may offer a means to stimulate the brain without a subject actually moving. These types of control conditions, which rule out stimulus artifact, will be extremely important in future studies.

It may also be possible ameliorate some of the interference problems “at source”. Here, except for removal of a small number of ferromagnetic screws, the VRHMD was essentially unmodified. Altered optics might enable the OLED screen to be moved further from the participant’s eyes, thereby reducing the impact of interference. Different screen types (e.g. LCD) might also generate less magnetic field, whilst lightweight magnetic screening might offer a means to contain magnetic fields within the headset itself; better still, the use of optical fibres might enable a VR headset without the need for a screen at all, and thus it might be possible to build a completely interference-free headset. Alternatively, different means to generate the VR environment, for example a CAVE type system, could theoretically be set up inside an MSR and would certainly offer an interference-free VR projection – albeit at the cost of a bespoke shielded room. Whilst these ideas offer a prospects for future technical development, this paper shows, for the first time, that even with relatively little modification to either VR or OPM-MEG, integration of these technologies feasible.

Finally, it is important to comment on the practicality of the system used. We found that participants did not complain of discomfort when using the 3D printed headcast in combination with the OPMs and VRHMD. However these were adult subjects who had all undergone neuroimaging experiments previously. It remains the case that the 3D printed helmet is heavy, and also whilst the OPMs themselves are quite light (4 g), the weight of cabling is heavy (33 g/m) and this cabling causes a torque on the subjects head. This, combined with the weight of the VRHMD means that this experimental set up may be impractical for some subject cohorts (e.g. particularly children). However, a new generation of commercial OPMs has recently become available (QuSpin.com) which are smaller (24.4mm in length compared to 110mm for first generation sensors), and their cabling lighter (3.3 g/m). The small nature of these new OPMs is likely to remove the need for heavy 3D printed helmets and significantly improve the practicality.

## 5. Conclusion

We have shown that OPM-MEG can be combined with virtual reality stimulation to deliver an immersive environment to a participant undergoing functional brain imaging. Unlike methods such as fMRI or conventional MEG, OPM-MEG allows movement during scanning which enables exploitation of the VR environment. Our initial results show that despite increased interference due to the VRHMD, we were able to measure both modulation of alpha-band oscillation by opening and closing the eyes, and the visual evoked field generated by displaying a reversing checkerboard in VR. Moreover, in a VR experiment in which a participant had to look around a wall to view a visual stimulus, we showed that MEG signals can be measured and that they map to expected areas of primary visual cortex. The significantly increased interference generated by the VRHMD remains a challenge for VR-OPM-MEG. Nevertheless, this technique could transform the type of experiment that can be undertaken using neuroimaging.

## Supplementary Material

Published Supplementary Documents
