# Supplementary material for: Towards OPM-MEG in a virtual reality environment
Source: Neuroimage. Author manuscript; Available in PMC 2021 Jul 13. (PMC8276767; doi:10.1016/j.neuroimage.2019.06.010)
Supplement: Published Supplementary Documents [file NIHMS1710582-supplement-Published_Supplementary_Documents.zip › 1-s2.0-S1053811919304951-mmc1.docx]

**SUPPLEMENTARY MATERIAL**


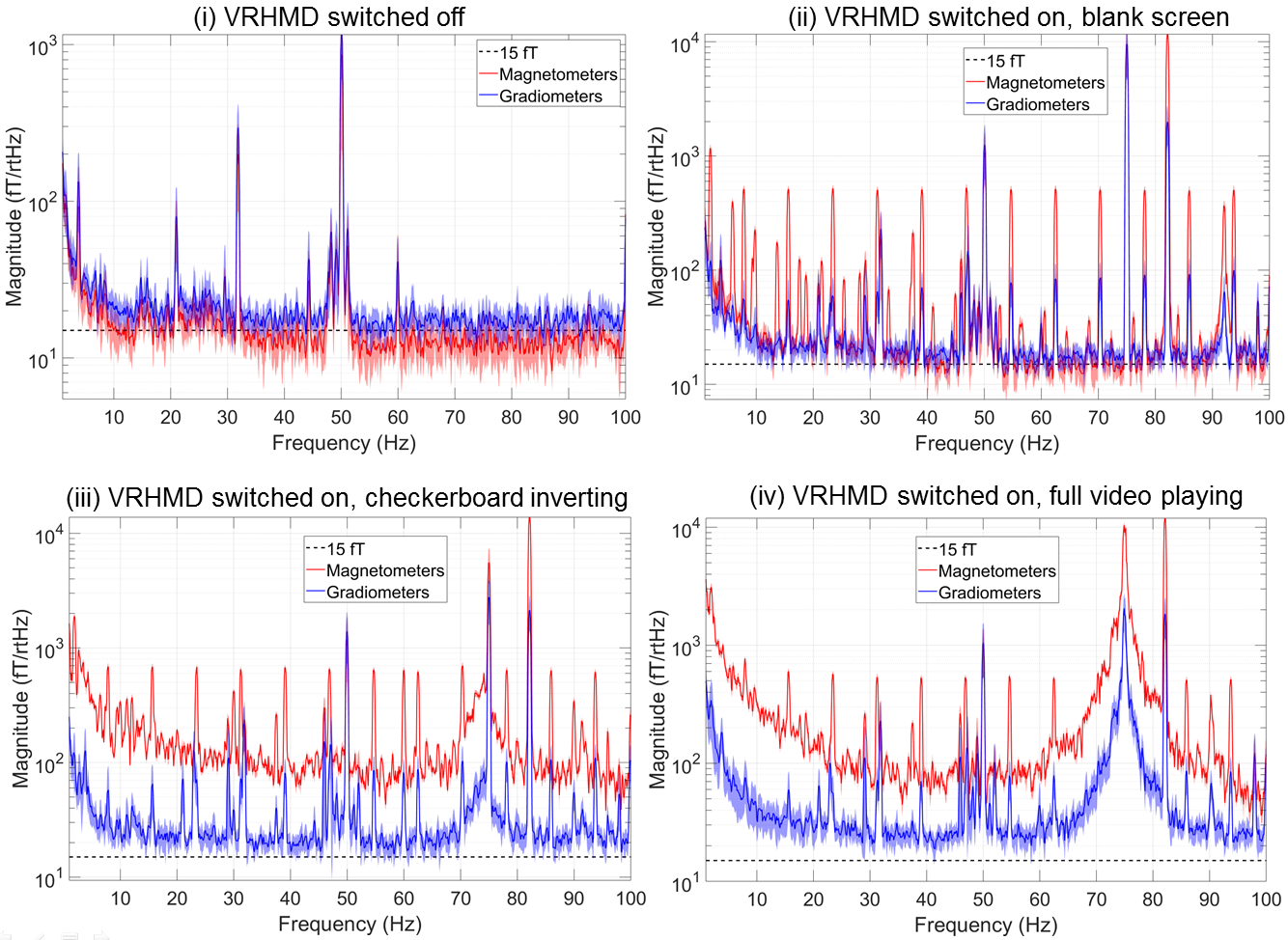


***Figure 6. Magnetic interference measured from the VRHMD.*** *Five sensors were placed in the scanner cast which was strapped to the back of a phantom. Ten planar gradiometers were synthesized from these sensors. Four 120-second recordings of the magnetic field were acquired, under the following conditions: (i) the VRHMD fully disconnected from its power source, (ii) the VRHMD switched on and displaying only a white screen, (iii) the VRHMD was switched on and displayed a version of the checkerboard experiment described, with no wall to occlude it, (iv) the VRHMD displayed a full-screen video at 60 frames per second. The mean amplitude for all sensors is shown in red, and for all gradiometers in blue. Note the ability of gradiometers to reject interference caused by the presence of the active VRHMD screen.*


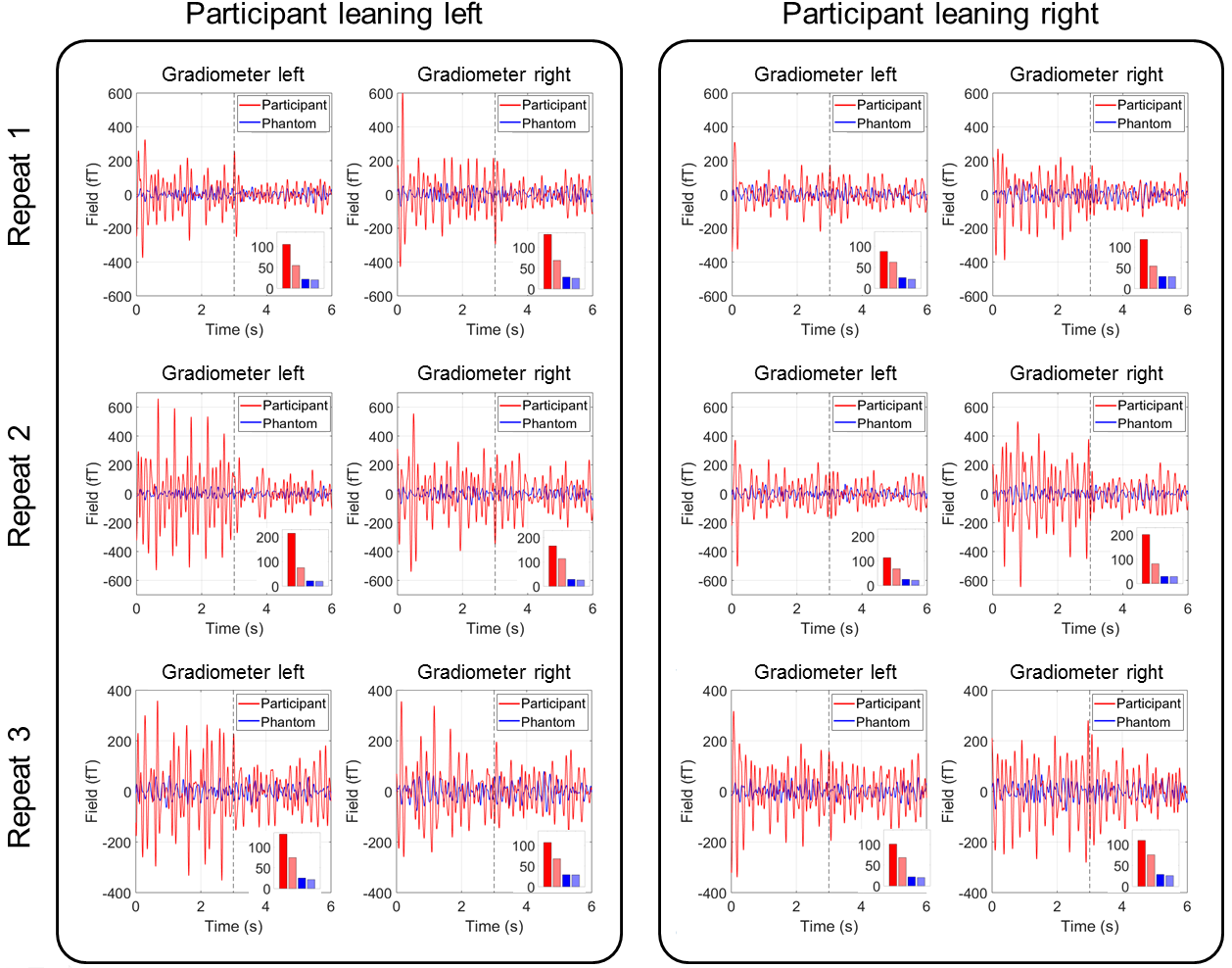


***Figure 7. All gradiometer pairs from the retinotopy experiment.*** *The experiment was repeated three times in the same participant. The result of stimulation with the checkerboard can be seen in the first three seconds, compared to the rest period from three to six seconds. Bar graphs showing standard deviations in fT are inset to the time courses: these are shown for the participant in the active window, the participant in the rest window, the phantom in the active window, and the phantom in the rest window in the following order: red, light red, blue and light blue.*


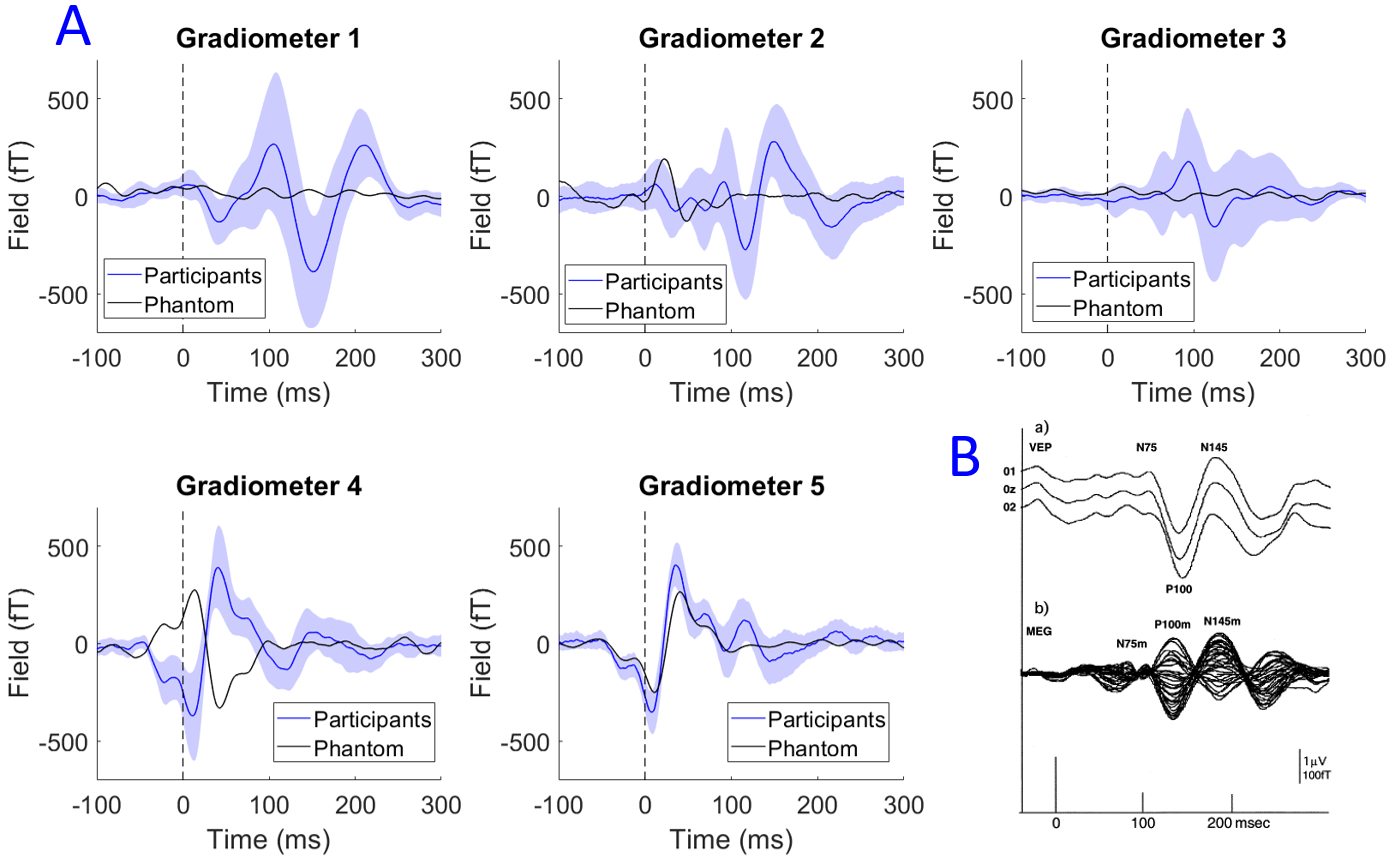


***Figure 8. Grand average of evoked responses from participants in experiment 2.*** *Panel A shows average responses (blue) in addition to the phantom (black). Standard deviation is shaded in light blue. A similar checkerboard evoked response from Shigeto et al. (1998) is shown in panel B.*
